# Use of Esketamine Nasal Spray in Patients with Treatment-Resistant Depression in Routine Practice: A Real-World French Study

**DOI:** 10.1155/2024/7262794

**Published:** 2024-07-16

**Authors:** Ludovic Samalin, Lila Mekaoui, Maud Rothärmel, Anne Sauvaget, Clotilde Wicart, Julien Dupin, Vanessa Cohignac, Emeline Gaudre-Wattinne

**Affiliations:** ^1^Department of Psychiatry, Clermont-Ferrand University Hospital, University of Clermont Auvergne, CNRS, IP, UMR 6602, Clermont-Ferrand, France; ^2^CMME, GHU paris psychiatrie et Neurosciences, Sainte Anne Hospital, Paris, France; ^3^University Department of Psychiatry, Therapeutic Excellence Center, Psychiatry Institute, Rouvray Hospital, Sotteville-lès-Rouen, France; ^4^Nantes University, Nantes University Hospital, Movement-Interactions-Performance, Nantes, France; ^5^Medical Affairs, Janssen-Cilag, Issy-les-Moulineaux, France

## Abstract

**Background:**

The efficacy and safety of esketamine nasal spray (ESK) were established in registration trials in patients with treatment-resistant depression (TRD). This French real-world study aimed to describe the treatment patterns, effectiveness, and safety of ESK in TRD patients over a 12-month follow-up.

**Materials and Methods:**

This study used secondary data from patient files of hospital-based psychiatrists and started during the first French patient early access to ESK. The response and remission rates with ESK were analyzed using the total score of the Montgomery–Åsberg Depression Rating Scale (MADRS). The time to first treatment response and work resumption were described (Kaplan–Meier method). Adverse events (AEs) were analyzed.

**Results:**

Prior to ESK initiation, the 157 analyzed patients (age ≤ 65 years, 82.8%; female, 66.2%) had depression for 10.5 years (median, IQR, 4.2–21.2) and received a median of 6 (3–8) previous treatment lines. At ESK initiation, the mean ± SD total MADRS score was 32.1 ± 7.7. At that time, ESK was combined with antidepressants (93.6% of patients; SNRI, 65.0%; SSRI, 57.3%) and/or other potentiation strategy (63.1%; atypical antipsychotics, 36.3%; lithium, 25.6%; antiepileptics, 21.7%). During the 12-month follow-up, 125 patients (79.6%) discontinued ESK. The median duration of ESK treatment was 19.4 weeks (IQR, 4.4–40.1). At 1 month after ESK initiation, 40.2% of still treated patients met criteria for clinical response and 19.7% for remission (median time to response, 5.7 weeks; 95% CI (4.1–8.4)). 82.6% of active patients were on sick leave at ESK initiation; the work resumption rate was 24% (13%–40%) 12 weeks later. AEs were reported in 68.6% of patients, serious AEs in 17.2%, and AEs leading to ESK discontinuation in 14.6%.

**Conclusion:**

These real-world effectiveness and safety data were consistent with findings from previous clinical trials, describing the real-life clinical experience of patients receiving ESK and confirming that ESK has its place in therapy for the treatment of TRD.

## 1. Introduction

Despite well-conducted treatments with antidepressants (AD) combined with psychotherapy for major depressive disorder (MDD), between 30% and 55% of patients experience treatment-resistant depression (TRD) [1, 2, 3, 4] defined as an inadequate response to at least two ADs with adequate dosing and duration. In France, out of 2.5 million people with depression, nearly 0.8 million have TRD [5]. According to a 2021 health barometer, 12.5% of people aged 18–85 had reported a major depression episode (MDE) within the last 12 months [6].

TRD is associated with poor quality of life, increased physical and psychiatric comorbidities, and elevated risk of suicide [4, 7]. In addition, in recent European and US studies involving large cohorts of patients with TRD, significant disease burden and healthcare costs were reported [8, 9, 10, 11].

The clinical efficacy and safety of esketamine (ESK) nasal spray, the S-enantiomer of ketamine, were demonstrated in combination with an oral AD in patients with TRD in double-blind placebo-controlled phase III studies (TRANSFORM 1–3 for induction treatment [12, 13, 14]; SUSTAIN 1 for maintenance treatment [15]; and SUSTAIN 2 [16] and SUSTAIN 3 [17] for long-term safety and efficacy up to 1 year and 6.5 years, respectively), and in a randomized, open-label, rater-blinded phase IIIb clinical trial comparing ESK with quetiapine (ESCAPE-TRD [18]).

Based on positive findings from the development program of ESK, in combination with a selective serotonin reuptake inhibitor (SSRI) or serotonin–norepinephrine reuptake inhibitors (SNRI), it was approved in March 2019 by the Food and Drug Administration, in conjunction with an oral AD, for the TRD treatment in adults [19] and in December 2019 by the European Medicines Agency for adults with treatment-resistant MDD, who have not responded to at least two different treatments with antidepressants in the current moderate to severe depressive episode [20]. Recently, expert recommendations were updated to include ESK within the available therapeutic arsenal for TRD [4]. Despite the scarcity of real-life data, the most recent ones showed that ESK was well-tolerated and effective in improving depression and anxiety in TRD [10, 21], notably in specific populations such as the elderly [22], patients with suicidal ideation or behavior [23], and with bipolar depression [24, 25], and suggested that comorbidities including substance use do not impact the drug effectiveness [21, 26]. However, ESK remains perhaps underused, probably due to several disease and treatment misconceptions as recently suggested [27]. Finally, few real-life data are available in Europe on the use of ESK in TRD patients.

The French ESKALE study aimed to describe the characteristics of the first TRD patients treated with ESK in France, the modalities of treatment use, the evolution of depressive symptoms, and the adverse events reported by TRD patients treated by ESK in real-world conditions over a 12-month follow-up period.

## 2. Materials and Methods

### 2.1. Study Design

ESKALE is a French, multicenter, noninterventional study based on secondary data from patient medical files.

Patient data were collected when available in patients' medical files over a 12-month period after ESK initiation, regardless if the treatment was maintained or not.

According to French legislation regarding secondary data use studies (research not involving the human person), the ESKALE study was conducted according the MR-004 reference method to ensure patient data confidentiality, and it was registered in the Health Data Hub (No. F20210125145040). All patients were informed about the study content before enrollment and had no objection to share their data.

### 2.2. Study Participants

**Figure 1:**
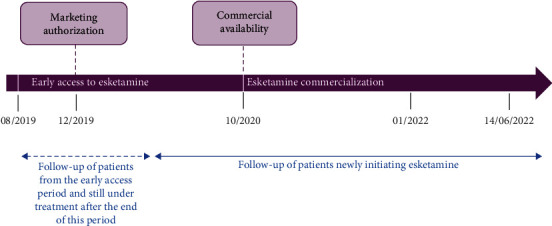
Study design.

Hospital-based psychiatrists included patients who initiated ESK between October 2019 (patients previously treated with ESK during the French early access period [28]) and July 2021 (after ESK was reimbursed [29]) (Figure 1). Eligible adult patients met criteria for TRD defined as nonresponse to at least two different treatments with antidepressants in the current moderate-to-severe depressive episode (according to the criteria of the Diagnostic and Statistical Manual of Mental Disorders, Fifth Edition, DSM-5 [30]). In addition, eligible patients could not receive electroconvulsive therapy (ECT) due to contraindication, no access to ECT, resistance to ECT, or patient refusal. Patients with ESK initiation over the early access program had to stay on treatment during this period to be included. Patients participating in interventional clinical trials within 30 days before ESK initiation or during the study treatment period could not be included.

### 2.3. Data Collection

At inclusion, upon initiation of ESK treatment, the following data were collected: patient and disease characteristics, medical pathway and history, previous treatments for MDD, use of ESK, and concomitant treatments for TRD. During follow-up (up to June 2022), data collection included the use of ESK and concomitant treatments and reason for discontinuation when applicable; sick leaves; disease assessment by the physician for depression severity when available (Montgomery–Åsberg Depression Rating Scale (MADRS)), and safety data.

### 2.4. Statistics

Considering the exclusively descriptive and noncomparative design of the ESKALE study, no formal sample size calculation was planned. However, for a binary endpoint with a 50% target frequency (i.e., the worst situation for the precision) and a confidence interval of 95%, a sample size of 150 patients gave a precision of 16%, considered as acceptable for a descriptive purpose.

Descriptive analysis was performed, using SAS® software (SAS Institute, North Carolina, USA), version 9.4. All available data were described at each study time point using standard descriptive statistics. The modalities of the use of ESK were described according to the age of patients at treatment initiation (<65 and ≥65 years). TRD lines prior to ESK initiation were defined as “well-conducted treatments” (treatment lines (monotherapy, combination, or neurostimulation) taken for at least 28 days [3], or at least six consecutive sessions of neurostimulation). The ESK effectiveness up to Month 12 was analyzed in patients still treated with ESK, on the basis of the total score of the 0–60 MADRS when available. The monthly proportion of responder patients (decrease in total MADRS score of at least 50% from treatment initiation) was described up to Month 12, as well as the remission rate (proportion of patients with a total MADRS score ≤10 points during at least two treatment administrations). In patients with treatment discontinuation, these analyses were performed using observed data and considering missing data as failure. The time to ESK response was estimated in patients still treated using the Kaplan–Meier method; the same method was used to estimate the rate of work resumption at Weeks 12, 24, and 48 in still treated patients on sick leave at ESK initiation.

## 3. Results

### 3.1. Baseline Characteristics

**Figure 2:**
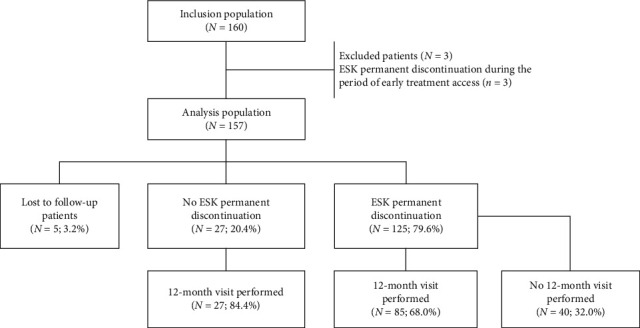
Disposition of patients. ESK, esketamine.

Of the 160 TRD treated patients included by 26 centers right across France, 157 were retained in the analysis population, after the exclusion of three patients previously registered in the early access program and who discontinued ESK within this period. Among these 157 analyzed patients, 112 (71.3%) completed the study (12-month follow-up) (Figure 2).

Baseline patient and disease characteristics are detailed in Table 1. The majority of patients were less than 65 years old (82.8%) and identified as female (66.2%). The median duration of depression was 10.5 years (interquartile range (IQR), 4.2; 21.2), experienced a median of 3 (1.0; 5.0) MDEs, and had at least one suicide attempt in 49.4% of the cases. Furthermore, the 144 evaluated patients had a mean baseline MADRS score of 32.1 ± 7.7, and 82.6% of patients who were employed (52/63) were on sick leave at ESK initiation.

**Figure 3:**
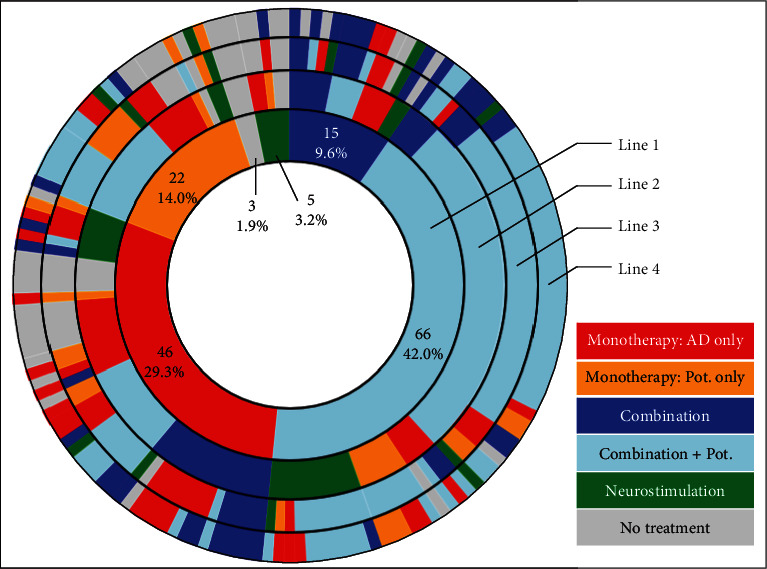
Chronological sequences of the four well-conducted treatment lines prior to esketamine initiation—sunburst representation. A well-conducted treatment line was defined as a treatment line (monotherapy, combination, or neurostimulation) taken for at least 28 days or at least six consecutive sessions of neurostimulation. This sunburst diagram allows to visualize the successive treatments of patients for each of the four previous well-conducted treatment lines being depicted by a concentric circle (hierarchical data). The circle in the center represents the first prior treatment line before esketamine initiation (i.e., previous line 1, innermost ring in the sunburst plot), with the hierarchy moving outward from the center. A segment of the inner circle bears a hierarchical relationship to those segments of the outer circle which lie within the angular sweep of the parent segment. On line 1, 43.3% of patients were treated with monotherapy (either one antidepressant (29.3%, *n* = 40) or one potentiation strategy (14.0%, *n* = 22)), 51.6% were treated with a combination of treatments (either a combination of antidepressants (9.6%, *n* = 15) and/or potentiation strategy(ies) (42.0%, *n* = 66)), and 3.2% (*n* = 5) received neurostimulation. On the previous line 2 (second circle), among the 66 patients (42.0%) with a combination of antidepressants and/or potentiation strategy(ies) on line 1, 41 patients (26.1%) received the same therapeutic strategy. They were 32 (20.4%) and 27 (17.2%) in this case for the lines 3 and 4, respectively. AD, antidepressant; Pot., potentiation.

Prior to ESK initiation, patients received a median of 6 (3; 8) previous “well-conducted” lines of treatment for their depression (≥3.0 lines: 88.5%). Previous treatments are detailed in Figure 3 for the four previous well-conducted treatment lines. During the current MDE, ESK was initiated after a median time of 19.1 months (IQR, 8.2; 43.3). Neurostimulation was performed in 71 patients (45.5%), or it was still considered for 53 of the 85 other patients (62.4%).

### 3.2. Treatment with ESK

At ESK initiation, 93.6% of patients were taking AD(s) (SNRI, 65.0%; SSRI, 57.3%), 6.4% did not receive any ADs, and 63.1% had potentiation strategy (second-generation antipsychotics, 36.3%; lithium, 25.5%; and antiepileptics, 21.7%) (Table 1). Almost all patients under 65 years of age (119/127, 93.7%) started ESK treatment at the dose of 56 mg, while the starting dose was 28 mg in 92.6% of those aged at least 65 (25/27). The 119 patients under 65 years of age further received an ESK dose of 84 mg in 80.7% of the cases (*n* = 96), after a median time of 8.5 days (IQR, 4.5–15.0−15.0). The 25 patients aged at least 65 (with a starting dose of 28 mg) received a dose of 56 mg in 88.0% of the cases (*n* = 22) after a median time of 4.5 days (3.0–9.0) and then a dose of 84 mg in 72.7% of the cases (*n* = 16) after a median time of 8.0 days (5.5–32.0). The first dose of ESK was administered in inpatient setting in 46.8% of the cases and ambulatory setting in 53.2% of patients, and the median duration of postadministration observation was 2 hr (IQR, 2.0; 2.0). Over the overall treatment period (median duration, 19.4 weeks; IQR, 4.4; 40.1), 122 patients (77.7%) reached the first treatment maintenance period (weeks 5–8) and 103 (65.6%) the second maintenance period (weeks 9–12). After a median weekly number of doses of 2.0 (IQR, 2.0; 2.0) during the induction period (weeks 1–4), it decreased during the maintenance phases: maintenance 1 : 1.0 (1.0; 1.2) and maintenance 2 : 0.6 (0.4; 0.8). A total of 59 patients (37.6%) discontinued ESK permanently after a 6-month treatment period, and 125 patients (79.6%) stopped it during the overall study follow-up (Figure 2), after a median time of 13.1 weeks (IQR, 4.1; 26.1). Main reasons given by the clinicians for discontinuation were unsatisfactory therapeutic effect (in 52.0% of the cases, after a median time of 5.4 weeks), in accordance to Summary of Product Characteristics (26.4%, after a median time of 28.9 weeks), patient demand (24.8%, after a median time of 9.6 weeks), and/or adverse events (8.8%, after a median time of 10.6).

### 3.3. Effectiveness

**Figure 4:**
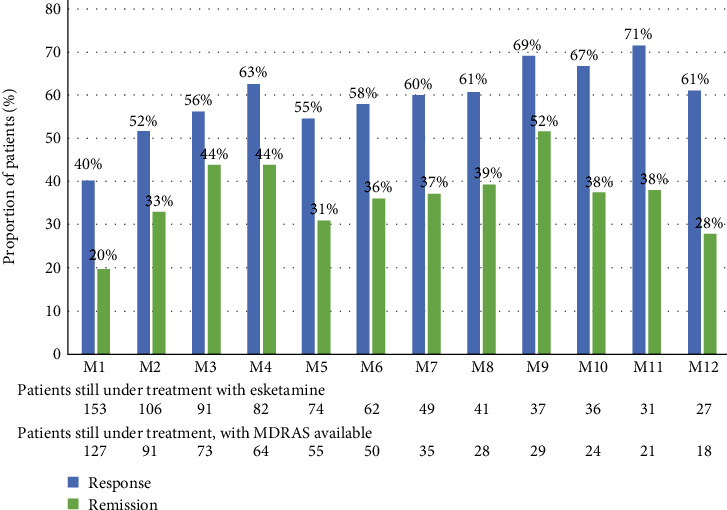
Effectiveness of esketamine up to Month 12. Analysis performed in patients still under treatment with esketamine and with MADRS available at each time point. Range of the MADRS score: from 0 to 60, a higher score indicating more severe depression. MADRS, Montgomery Asberg Depression Rating Scale. Response to esketamine, decrease in total MADRS score of at least 50% from treatment initiation; remission, total MADRS score at 10 points maximum during at least two treatment administrations.

Among the 144 patients (91.7%) who completed the MADRS at baseline and met criteria to be included in the response analysis, 93 patients (64.6%) reached a clinical response during the 12-month follow-up period, after a median time of 3.1 weeks (IQR, 1.1;5.7). Using the Kaplan–Meier method, the median time to the response with ESK was estimated at 5.7 weeks (95% CI (4.1–8.4)) among the 144 evaluable patients (Figure 4).

**Figure 5:**
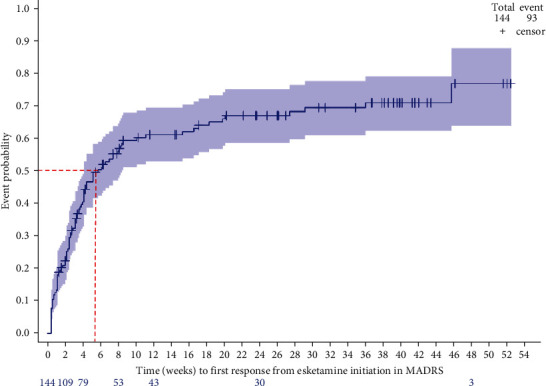
Time to esketamine response—Kaplan–Meier analysis. MADRS, Montgomery–Asberg Depression Rating Scale. Response to esketamine: decrease in total MADRS score of at least 50% from treatment initiation.

At the end of the induction phase (Month 1), 40.2% of patients still receiving ESK (51/127) reached a clinical response to treatment, and 19.7% (25/127) were in remission from MDE (Figure 5). These proportions improved at Month 3: 56.2% (41/73) and 43.8% (32/73), respectively. Using the Kaplan–Meier method, the response rates were 40% (95% CI (33%–49%)) and 61% (53%–69%), at 1 and 3 months, respectively (Figure 4).

At ESK permanent discontinuation, the MADRS score was available for 92 of the 125 patients who discontinued treatment (73.6%). At that time, the response and remission rates were 45.7% (42/92) and 35.9% (33/92), respectively. Considering missing data as failure, these respective proportions were 33.6% (42/125) and 26.4% (33/125).

**Figure 6:**
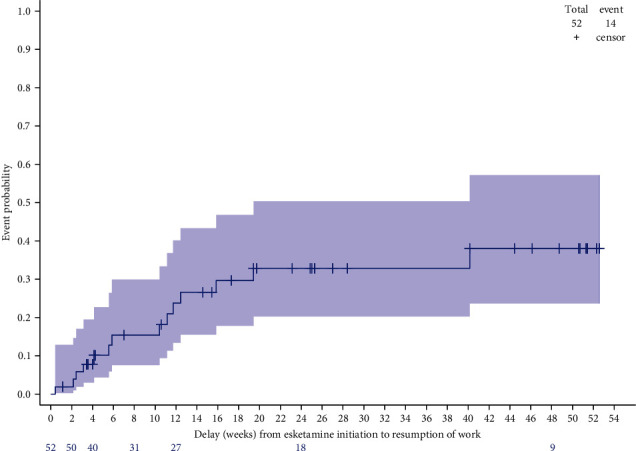
Time to work resumption in patients on sick leave at esketamine initiation—Kaplan–Meier analysis.

Using the Kaplan–Meier method, 24% (95% CI (13%–40%)) of patients with professional activity and on sick leave at ESK initiation resumed to work 12 weeks later, 33% (20%–50%) 24 weeks later, and 38% (24%–57%) 48 weeks later (Figure 6).

### 3.4. Safety

Over the study period, at least one adverse event (AE) was reported in 66.2% of patients, mainly as dissociation (34.4%), somnolence (15.9%), vertigo (15.9%), sedation (14.6%), blood pressure increase (14.0%), and anxiety (14.0%) (Table 2). Among these 104 patients, 90 patients (86.5% and 57.3% of all patients) experienced AEs assessed as related to ESK according to the clinician's judgment, including all the patients who suffered from dissociation, somnolence, vertigo, and sedation. For one patient (0.6%), an ESK dependence with moderate severity was reported.

Overall, 26 patients (16.6%) presented with at least one serious event, including 21 patients (13.4%) with psychiatric disorders (notably 10 patients (6.4%) who experienced worsening of depression, five patients (3.2%) with suicidal ideation, two patients (1.3%) with mental disorder, and one patient (0.6%) with suicidal attempt) (Table 3). Among these 26 patients, six patients (23.1% and 3.8% of all patients) had ESK-related SAE(s), including two patients (1.3%) with suicidal ideation, one patient (0.6%) with suicidal attempt, one patient (0.6%) with worsening of depression, and one patient (0.6%) with mental disorder.

AEs leading to ESK permanent discontinuation were experienced by 21 patients (13.4%) with, in particular, blood pressure increase (*n* = 6), somnolence (*n* = 5), dissociation (*n* = 4), vertigo (*n* = 4), suicidal ideation (*n* = 2), and suicidal attempt (*n* = 1). One fatal nonrelated event (from unknown cause) was reported during patient follow-up.

## 4. Discussion

ESKALE is one of the first studies generating real-world evidence in Europe on TRD patients treated with ESK and followed over a 12-month period. It provides new information on the treatment use, effectiveness, and safety in routine clinical practice.

### 4.1. Patient Clinical Characteristics at Treatment Initiation

When TRD patients started ESK treatment, they suffered from MDD for more than 10 years in median, experienced a median of three major depressive episodes (MDE) within their lifetime, and had at least one suicide attempt in nearly half of them. Even if their mean MADRS score was slightly lower than the score reported in previous clinical trials (32 *versus* 37) [12, 13], they already received a median of six previous lines of treatment for their depression (≥3.0 lines, 88.5%), and neurostimulation was often used prior to ESK (or previously considered). In the phase III TRANSFORM-2 trial, TRD patients had a higher mean total MADRS score (37.0) at baseline and received less treatment lines prior to ESK with AD initiation (≥3.0 lines, 31.6%) [13]. In the ESCAPE-TRD trial, a median of 3.4 MDE was reported prior to ESK initiation, with a mean total MADRS score of 31.4 at baseline, but also a lower number of previous lines of treatment (≥3, 39.3%) [16], highlighting the complexity of the profile of the TRD patients followed in the ESKALE study in a real-life setting.

### 4.2. Modalities of use of Esketamine

The use of ESK was in accordance with its Summary of Product Characteristics [20]. In particular, the drug was prescribed in patients with normal blood pressure, the starting dose depended on patient age (56 mg if <65 years and 28 mg if ≥65 years) with a possible dose increase up to 84 mg, the drug was administered twice a week within the first 4 weeks, and then the frequency decreased over the maintenance phase of the treatment; ESK was mostly combined with SSRI, SNRI, and potentiation strategy. However, a minority of patients (6.4%) did not receive any ADs in combination with ESK.

### 4.3. Esketamine Effectiveness

In the ESKALE study, results on the effectiveness of ESK showed that TRD patients improved under ESK as soon as Month 1, i.e., at the end of the induction phase (clinical response and remission rates: 40.2% and 19.7%, respectively). Other real-life French clinical data from 50 patients included in a single institution showed a response rate of 52.4% and a remission rate of 38.1% around 28 days (remission defined as a total MADRS score ≤12 points *versus* ≤10 points in the ESKALE study) [31]. In a recent retrospective observational Italian study (REAL-ESK), this improvement was lower within the first month of treatment (28.4% and 11.2%, respectively), using the same definitions as for the ESKALE study [21], possibly because a lower number of patients were prescribed the 84 mg dosage within the first 4 weeks of treatment (REAL-ESK, 26.5%; ESKALE, 59.4%) despite a slightly more severe disease at ESK initiation (mean depression duration, 19 ± 11 *versus* 15 ± 13; mean MADRS score at 35 ± 9 *versus* 32 ± 8). At Month 3, the clinical response and remission rates improved in both studies and were broadly similar (ESKALE, 56.2% and 43.8%, respectively; REAL-ESK, 64.2% and 40.6%). The response rates at Month 3 were 57% and 69% under ESK in two other Italian noninterventional studies involving 29 and 149 treated patients, respectively [32, 33], and a machine learning approach identified anhedonia and anxiety as predictive factors of response to ESK [33]. Furthermore, a reduction of depressive symptoms was reported within the first month and perpetrated during 48 weeks of maintenance in the SUSTAIN-1 clinical trial [15]. In the ESCAPE-TRD trial, the absolute rate of remission was 27.1% at Week 8 [18]. In addition, in the ESKALE study, most (82.6%) of patients with professional activity were on sick leave at ESK initiation, and about a quarter of these patients have returned to work as early as 12 weeks after starting treatment with esketamine.

### 4.4. Esketamine Discontinuations

Over patient follow-up, we observed a high rate of ESK discontinuation (80%), mainly due to unsatisfactory therapeutic effect (in 52.0% of the cases) according to psychiatrists. These proportions were higher than those reported up to Week 32 in the ESCAPE-TRD trial (23.2% of patients discontinued treatment, due to lack of treatment efficacy in 8.2% [18]). In the ESKALE study, treatment discontinuations for unsatisfactory therapeutic effect occurred after a median time of 5.4 weeks, i.e., after the induction period which is consistent with the SmPC recommendations (“evidence of therapeutic benefit should be evaluated at the end of induction phase to determine need for continued treatment” [20]). In addition, the constraint for patients to be treated at hospital could lead them to stop ESK earlier than for a drug administered at home.

However, the response and remission rates remained high at the time of treatment discontinuation (45.7% and 35.9%, respectively, using observed data and 33.6% and 26.4% considering missing data as failure). In a systematic review of randomized double-blind controlled-placebo studies, it was shown that ESK had a significantly higher rate of discontinuation due to intolerability [34]. Based on the ESK summary of product characteristics, treatment is recommended for at least 6 months after depressive symptoms improve [20]. However, questions persist/remain on treatment duration and discontinuation in a real-life setting. Recently published consensus-based guidelines recommend a treatment duration of at least 6 months for patients with satisfactory clinical response. With regard to treatment discontinuation, experts recommend to preferentially stop treatment when administered once every 2 weeks, with no change in dose required [35].

### 4.5. Esketamine Tolerability

The safety profile of ESK was consistent with the Summary of Product Characteristics [20], and only one dependence to ESK was reported. In addition, as previously reported in clinical trials [12, 13, 14, 15] as well in the Italian noninterventional REAL-ESK [21], adverse events related to ESK mainly included dissociation, somnolence, sedation, and blood pressure increase, but few of these events led to ESK discontinuation (dissociation, 4 patients; somnolence, 5; and blood pressure increase, 6). Based on the now established safety profile of ESK after data collected during the previous phase III, IIIb, and long-term clinical trials [12, 13, 14, 15, 16, 17, 18], no new safety signals were observed over patient follow-up in the real-world ESKALE study (median treatment duration of 19.4 weeks). In particular, while questions were initially raised on the long-term safety profile of ESK [36], similar safety in long-term as shorter studies was recently provided from the SUSTAIN-3 clinical trial, after a median treatment duration of 37.7 months [17]. In addition, final findings from the ESCAPE-TRD trial comparing esketamine with quetiapine, an active comparator frequently used in TRD patients, showed no new safety signals over a 32-week long follow-up [18].

### 4.6. Study Limitations

Our study had some limitations, mainly due to its noninterventional design (no comparator, assessments available only when performed in routine clinical practice, and data available only when previously recorded in patients' medical files). In particular, the clinical assessment of depression, using the MADRS, was not performed at each study time point that could limit the interpretation of our results. In addition, MADRS analysis should be interpreted with caution considering the number of missing questionnaires for patients still treated (17.0% of missing data at Month 1% and 19.8% at Month 3), which can lead to an overestimation of our effectiveness results. It should be noted that the narcotic status and the hospital restricted access of the drug in France could also have had an impact on patient recruitment and characteristics, as well as potential treatment misconceptions by clinicians as recently suggested [27]. Furthermore, some of the analyzed patients were included during the French early access program to ESK, with more selection criteria than in real-life conditions of prescription after its commercial availability. It should also be noted that physicians may have a scarce drug experience at the beginning of ESK availability which could led to treat more severe patients. In addition, the identification of patients who have stopped the treatment early and their evolution after treatment discontinuation could add complementary information of interest. Finally, even if the ESK safety profile observed in the ESKALE study was consistent with findings from previous clinical trials, an under-reporting of AEs usually observed in noninterventional studies cannot be excluded.

## 5. Conclusions

Our positive findings are even more important that TRD is related with a heavy burden (lower patient's health and more hospitalizations unrelated to depression during MDE) [37]. However, the fact that the funding of mental health institutions relies on an annual budget allocation in France could hinder access to therapeutic innovation and new medication due to long length of stay [38, 39]. In this context, the risk of altering the continuum of care in psychiatry does raise the question on the optimization of the funding of further innovative medicine in that field where unmet medical needs are still significant.

These real-life data on the first 157 patients treated with ESK for TRD in France were consistent with previous findings from research clinical trials. These data allowed to describe patient profiles benefiting from ESK in France, as well as its modalities of use, effectiveness, and tolerance in routine medical practice, thereby showing that ESK could be considered as a therapeutic alternative in the treatment of TRD patients. However, some patients did not respond to ESK when used in real-life conditions in the ESKALE study. Further exploratory studies designed to search for the prognostic factors for treatment response would help to identify the TRD patients most likely to benefit from ESK.

## Figures and Tables

**Table 1:** Characteristics of patients with treatment-resistant depression at initiation of esketamine.

Characteristics	Number of analyzed patients	Total (*N* = 157)
Demographics and physical examination		
Age (years)	157	—
Mean (SD)	—	49.1 (15.8)
<65 years, *n* (%)	—	130 (82.8%)
Female patient, *n* (%)	157	104 (66.2)
Normal blood pressure, *n* (%)	140	138 (94.5%)
Depression history		
Duration of depression (years), median (IQR)	137	10.5 (4.2; 21.2)
Total number of MDE (including the current episode), median (IQR)	127	3.0 (1.0; 5.0)
Medical history, *n* (%)		
Anxiety disorders	141	40 (28.4)
Drug or alcohol abuse	155	24 (15.5)
Posttraumatic stress disorder	141	21 (14.9)
Suicide attempt(s)	156	77 (49.4)
Well-conducted lines of treatment *⁣*^*∗*^ prior to esketamine initiation		
At least one, *n* (%)	157	154 (98.1)
≥3, *n* (%)	157	139 (88.5)
Median number (IQR)	154	6.0 (4.0; 9.0)
Current major depressive episode		
Severe episode, *n* (%)	154	116 (75.3)
Clinical subtype, *n* (%)	136	—
Anxiety features	—	44 (32.4)
Melancholic features	—	22 (16.2)
Psychotic features	—	7 (5.1)
Atypical features	—	7 (5.1)
Catatonic features	—	1 (0.7)
No subtype identified	—	49 (36.0)
Clinical assessment at esketamine initiation, mean (SD)		
Total MADRS score^&^	144	32.1 (7.7)
Patient with professional activity at esketamine initiation	156	65 (41.7)
On sick leave at esketamine initiation	63	52 (82.6)
Pharmacologic treatments at esketamine initiation (*N* = 157), *n* (%)	157	154 (98.1)
Antidepressants	—	147 (93.6)
SNRI	—	102 (65.0)
SSRI	—	90 (57.3)
NaSSA	—	80 (51.0)
TCA	—	41 (26.1)
MAOI	—	4 (2.5)
Others	—	36 (22.9)
Potentiation strategy	—	99 (63.1)
Second generation antipsychotic^¶^	—	57 (36.3)
Lithium	—	40 (25.6)
Antiepileptic	—	34 (21.7)
Thyroid hormone	—	13 (8.3)
Dopaminergic agonist	—	6 (3.8)
Folic acid	—	1 (0.6)
Other pharmacologic treatments	—	69 (43.9)
Anxiolytic	—	50 (31.8)
Hypnotic	—	27 (17.2)
First-generation antipsychotic	—	25 (15.9)
Neurostimulation during the current MDE (*N* = 156), *n* (%)	—	71 (45.5)
Neurostimulation techniques	60	—
Electroconvulsive therapy	—	37 (61.7)
Repetitive transcranial magnetic stimulation	—	37 (61.7)
Transcranial direct current stimulation	—	2 (3.3)

CGI-SS-r, Clinical Global Impression of Severity of Suicidality-revised; GAF, Global Assessment of Functioning; IQR, interquartile range; MADRS, Montgomery–Asberg Depression Rating Scale; MAOI, monoamine oxidase inhibitor; MDD, major depression disorder; MDE, major depression episode; NaSSA, noradrenergic and specific serotonergic antidepressant; PHQ-9, Patient Health Questionnaire; SD, standard deviation; SNRI, serotonin and norepinephrine reuptake inhibitor; SSRI, selective serotonin reuptake inhibitor; TCA, tricyclic antidepressant. *⁣*^*∗*^A previous well-conducted treatment line was defined as a treatment line (monotherapy, combination, or neurostimulation) taken for at least 28 days or at least six consecutive sessions of neurostimulation. ^&^Range of the MADRS score, from 0 to 60, a higher score indicating more severe depression; range of the CGI-SS-R score, from 0 (normal, not at all suicidal) to 6 (among the most extremely suicidal patients); range of the PHQ-9 score, from 0 (no depression) to 27 (most severe depression); range of the GAF, from 100 (extremely high functioning) to 1 (severely impaired). ^¶^Quetiapine (*n* = 26, 16.6%), clozapine (*n* = 5, 3.2%), risperidone (*n* = 3, 1.9%), olanzapine (*n* = 3, 1.9%), aripiprazole (*n* = 3, 1.9%), sulpiride (*n* = 1, 0.6%), and not specified (*n* = 24, 15.6%).

**Table 2:** Adverse events (AEs) and AEs assessed as related to esketamine (≥2% of patients).

System organ class/preferred term	Total (*N* = 157)					
	AEs			Related AEs		
	Nb AEs	Nb pat	% pat	Nb AEs	Nb pat	% pat
Any AEs	889	104	66.2	811	90	57.3
Psychiatric disorders	411	81	51.6	354	66	42.0
Dissociative disorder	294	54	34.4	294	54	34.4
Anxiety	36	22	14.0	30	18	11.5
Worsening of depression	16	15	9.6	5	4	2.5
Suicidal ideation	10	9	5.7	5	5	3.2
Nervous system disorders	266	43	27.4	263	42	26.8
Somnolence	104	25	15.9	104	25	15.9
Sedation	81	23	14.6	81	23	14.6
Altered pitch perception	45	15	9.6	45	15	9.6
Paresthesia	14	6	3.8	14	6	3.8
Headache	6	4	2.5	6	4	2.5
Ear and labyrinth disorders	74	26	16.6	74	26	16.6
Vertigo	64	25	15.9	64	25	15.9
Investigations	68	24	15.3	63	20	12.7
Blood pressure increased	64	22	14.0	62	20	12.7
Gastrointestinal disorders	25	12	7.6	24	11	7.0
Nausea	14	10	6.4	14	10	6.4
Vomiting	9	4	2.5	9	4	2.5
General disorders and administration site conditions	12	8	5.1	9	5	3.2
Asthenia	6	5	3.2	4	3	1.9
Eye disorders	10	4	2.5	10	4	2.5
Respiratory, thoracic, and mediastinal disorders	4	4	2.5	4	4	2.5
Vascular disorders	3	3	1.9	3	3	1.9
Injury, poisoning, and procedural complications	5	2	1.3	0	0	0.0
Skin and subcutaneous tissue disorders	2	2	1.3	2	2	1.3
Cardiac disorders	1	1	0.6	1	1	0.6
Endocrine disorders	1	1	0.6	0	0	0.0
Metabolism and nutrition disorders	1	1	0.6	0	0	0.0
Musculoskeletal and connective tissue disorders	1	1	0.6	1	1	0.6
Neoplasms benign, malignant, and unspecified (incl cysts and polyps)	1	1	0.6	0	0	0.0
Renal and urinary disorders	1	1	0.6	3	1	0.6

Nb, number; Pat, patient.

**Table 3:** Serious adverse events (SAEs).

System organ class/preferred term	Total (*N* = 157)					
	SAEs			Related SAEs		
	Nb SAEs	Nb pat	% pat	Nb SAEs	Nb pat	% pat
Any SAEs	32	26	16.6	7	6	3.8
Psychiatric disorders	23	21	13.4	5	5	3.2
Worsening of depression	10	10	6.4	1	1	0.6
Suicidal ideation	5	5	3.2	2	2	1.3
Mental disorder	2	2	1.3	1	1	0.6
Anhedonia	1	1	0.6	0	0	0.0
Anxiety	1	1	0.6	0	0	0.0
Confusional state	1	1	0.6	0	0	0.0
Flat affect	1	1	0.6	0	0	0.0
Suicidal behavior	1	1	0.6	0	0	0.0
Suicide attempt	1	1	0.6	1	1	0.6
General disorders and administration site conditions	1	1	0.6	0	0	0.0
Death	1	1	0.6	0	0	0.0
Injury, poisoning, and procedural complications	5	2	1.3	0	0	0.0
Fall	2	2	1.3	0	0	0.0
Ankle fracture	1	1	0.6	0	0	0.0
Clavicle fracture	1	1	0.6	0	0	0.0
Pelvic fracture	1	1	0.6	0	0	0.0
Investigations	1	1	0.6	0	0	0.0
Blood pressure increased	1	1	0.6	0	0	0.0
Neoplasms benign, malignant, and unspecified (incl cysts and polyps)	1	1	0.6	0	0	0.0
Bladder cancer recurrent	1	1	0.6	0	0	0.0
Respiratory, thoracic, and mediastinal disorders	1	1	0.6	1	1	0.6
Throat irritation	1	1	0.6	1	1	0.6

Nb, number; Pat, patient.

## Data Availability

The datasets supporting the conclusions of this article are available from the corresponding author upon reasonable request.
